# Use of Indigenous *Hanseniaspora vineae* and *Metschnikowia pulcherrima* Co-fermentation With *Saccharomyces cerevisiae* to Improve the Aroma Diversity of Vidal Blanc Icewine

**DOI:** 10.3389/fmicb.2018.02303

**Published:** 2018-10-22

**Authors:** Bo-Qin Zhang, Jing-Yun Shen, Chang-Qing Duan, Guo-Liang Yan

**Affiliations:** ^1^Centre for Viticulture and Enology, College of Food Science and Nutritional Engineering, China Agricultural University, Beijing, China; ^2^Key Laboratory of Viticulture and Enology, Ministry of Agriculture, Beijing, China

**Keywords:** *Hanseniaspora vineae*, *Metschnikowia pulcherrima*, *Saccharomyces cerevisiae*, vidal icewine, mixed fermentation, diversified aroma profile

## Abstract

Using novel non-*Saccharomyces* strains is regarded as an effective way to improve the aroma diversity of wines to meet the expectations of consumers. The non-*Saccharomyces* Hanseniaspora vineae and *Metschnikowia pulcherrima* have good aromatic properties useful for the production of table wine. However, no detailed information is available on their performances in icewine fermentation. In this study, simultaneous and sequential fermentation trials of indigenous *M. pulcherrima* CVE-*MP*20 or *H. vineae* CVE-*HV*11 with *S. cerevisiae* (*SC*45) were performed in 50-L fermenters of Vidal icewine, respectively. The results showed that *SC*45 cofermented with different non-*Saccharomyces* strains could generate a distinct aroma quality of icewine compared with four *S. cerevisiae* strain monocultures as evidenced by principal component analysis (PCA). Mixed fermentation of *MP*20/*SC*45 produced higher contents of acetate esters and β-damascenone with lower C_6_ alcohols relative to *SC*45 monoculture. Interestingly, *HV11*/*SC*45 generated the highest amounts of C_6_ alcohols [(Z)-3-hexen-1-ol and (E)-3-hexen-1-ol], higher alcohols (isobutanol, isopentanol, and 2-phenylethanol), acetate esters (2-phenethyl acetate and isoamyl acetate), *cis*-rose oxide, β-damascenone, and phenylacetaldehyde. Compared with simultaneous inoculation, sequential inoculation could achieve higher aroma diversity and produce higher intensity of fruity, flowery, and sweet attributes of icewine as assessed by calculating the odor activity values (OAVs). Our results verified the desired enological characteristics of *H. vineae* strain in icewine fermentation and also demonstrated that using indigenous non*-Saccharomyces* and *Saccharomyces* strains is a feasible way to improve aroma diversity of icewine products, which could provide an alternative way to meet the requirement of wine consumers for diversified aromatic quality.

## Introduction

Icewine is a dessert wine that is made from grapes, which have been left on the vine until weather conditions are cold enough to freeze the grapes. During freezing, most of the water in grape berries is consequently frozen, and sugars, acids, and nitrogenous compounds are concentrated (Chamberlain et al., 1997). To produce an authentic icewine, the entire harvesting and pressing process must be carried out below −8°C, and the juice sugar content must be >35 Brix at the time of pressing (VQA Ontario, 2014^1^). Vidal Blanc and Riesling are two main cultivars to produce icewine due to their relatively higher resistance to harsh conditions experienced by grapes prior to picking (Khairallah et al., 2016). Icewine contains higher and distinct volatile compounds relative to table wines, which largely determine the quality of icewine products (Bowen and Reynolds, 2015). The *S. cerevisiae* plays a major impact in the formation of icewine aromatic profiles because it produces a number of aroma compounds including higher alcohols, esters, fatty acids, and carbonyl compounds during alcoholic fermentation. Compared to table wine yeast, icewine yeast is challenged by more extreme stresses during alcoholic fermentation especially high sugar concentrations (above 35 Brix) and low fermentation temperature (15–18°C), which lead to reduced cell growth, prolonged fermentation time, and lower-than-desired alcohol levels (Kontkanen et al., 2004). These stresses can also induce significant changes of yeast metabolism and the production of metabolites including acetic acid, glycerol, and aromatic compounds, and ultimately affect icewine quality (Heit et al., 2018). Several works have confirmed that different commercial *S. cerevisiae* strains could produce diversified aromatic profiles during icewine fermentation and lead to different sensory characteristics (Erasmus et al., 2004; Crandles et al., 2015; Synos et al., 2015).

In recent years, the consumer requirements for distinctive aromatic characteristics urge winemakers to develop a variety of ways to manipulate specific aroma compounds and increase the complexity of wine. In this context, non-*Saccharomyces* wine yeasts have received more attention because these strains have several desired enological characteristics that are absent in *S. cerevisiae*, such as producing high levels of aroma compounds and producing, and secreting several enzymes (esterases, ß-glycosidases, lipases, and proteases) (Andorrà et al., 2012; Jolly et al., 2014). Cofermentations of *S. cerevisiae* with selected non-*Saccharomyces* yeasts in a controlled manner have proven to produce distinct aroma profiles and improve the complexity of table wine (Andorrà et al., 2010; Anfang et al., 2010; Jolly et al., 2014; Cañas et al., 2015). Among these non-*Saccharomyces* yeast species, *Metschnikowia pulcherrima* is often used to reduce alcohol of wine by sequential inoculation with *S. cerevisiae* (Varela et al., 2017). Cofermentation with *Saccharomyces uvarum* and this yeast can improve sensory attributes of wine by increasing the concentrations of 2-phenylethanol and 2-phenylethyl acetate (Varela et al., 2016), and a positive sensory contribution to wine was also found for Chenin Blanc in sequential fermentations (Jolly et al., 2017b). However, it was also reported that using the same inoculation method for Chardonnay can produce an inferior quality compared with fermentation using *S. cerevisiae* (Jolly et al., 2017a). *Hanseniaspora vineae* is a controversial non-*Saccharomyces* species belonging to *Hanseniaspora* spp., that is considered an apiculate yeast due to its cell morphology (Lleixà et al., 2016). Two earlier studies discouraged the development of apiculate yeasts in winemaking since the fermentation using their strains resulted in the production of large amounts of ethyl acetate and acetic acid (Velázquez et al., 1991; Ciani and Picciotti, 1995). However, inconsistent results have also been reported. Viana et al. (2011) and Medina et al. (2013) found that sequential fermentation of *S. cerevisiae* with *H. vineae* could increase fruity aromas and produce a high amount of acetate esters, such as 2-phenylethyl acetate and ethyl acetate, in the fermentations of Tempranillo and Chardonnay, respectively. These results suggested that the enological effect of non-*Saccharomyces* in mixed fermentation is largely dependent on inoculated species/strains, must composition, and fermentation conditions.

The nutrition status of must significantly influences aromatic profiles of wine products (Bell and Henschke, 2010). Considering the specific environment needed for icewine fermentation, it is interesting to investigate the effects of the multiculture of non-*Saccharomyces* and *S. cerevisiae* strains on aroma characteristics of icewine. However, until now, little information is available. Using autochthonous or locally selected wine yeasts is increasingly encouraged in wine-making because these yeasts can adapt well to the micro-conditions of the wine region and generate peculiar aromatic notes, which impart the wine with typical sensory characteristics specific to each wine area (Calabretti et al., 2012; Liu et al., 2016; Tofalo et al., 2016). The icewine industry in China has developed rapidly in recent years, and China has become an important icewine producer (Tang et al., 2013; Lan et al., 2016). Huairen is a typical icewine production region in the northeast of China with a suitable climate for icewine making (Huang et al., 2018). At present, most Chinese wineries use imported commercial yeast strains to inoculate icewine fermentation, which can reduce the variability of autochthonous yeast strains and thus, negatively influence the icewine aroma complexity and regional characteristics. In our previous work, two potential non-*Saccharomyces* wine strains *M. pulcherrima* CVE-*MP*20 and *H. vineae* CVE-*HV*11 had been isolated from spontaneous fermentation of Vidal icewine in Huairen. The preliminary experimental results showed that they have good technological characteristics and present potential application in icewine-making. To improve enological characteristics of icewine through the use of indigenous non-*Saccharomyces* strains, the effects of mixed fermentation of indigenous *M. pulcherrima MP*20 and *H. vineae HV*11 with indigenous icewine *S. cerevisiae* CVE-*SC*45 (simultaneous and sequential inoculation) on aromatic profiles of Vidal icewine were investigated in a 50 L fermenter, respectively in this study. Besides CVE-*SC*45 monoculture, the other three *S. cerevisiae* wine yeasts (one indigenous yeast strain CVE-*SC*42 and two commercial wine yeast strains XR and R2) were used as reference monoculture strains. The cell growth, physicochemical products, and aromatic compounds of icewine after alcoholic fermentation with different inoculations were determined and compared.

## Materials and methods

### Yeast strains

Four autochthonous strains, including two *S. cerevisiae* strains (CVE-*SC*42 and CVE-*SC*45) and two non-*Saccharomyces* strains (*M. pulcherrima* CVE-*MP*20 and *H. vineae* CVE-*HV*11), were used in the present study. They were isolated from 10-L spontaneous fermentation of Vidal grape juice with initial addition of 80 mg/L SO_2_ and fermentation under 15–17°C (Unpublished results). The determinations of technological characteristics showed that both non-*Saccharomyces* strains had high tolerance to ethanol, SO_2_, and sugar, low production of H_2_S, and high production of protease and glucosidase (Table S1). The four strains were deposited in the China General Microbiological Culture Collection Center (CGMCC), and the preservation numbers are 42-161017 (CVE-*SC*42), 45-161017 (CVE-*SC*45), 9-161017 (*M. pulcherrima* CVE-*MP*20), and 4-161017 (*H. vineae* CVE-*HV*11). Two commercial *S. cerevisiae* strains XR and R2 (Lallemand, France) were applied as reference strains as well. These strains were stored at −80°C in YPD medium with the addition of glycerol (20% v/v final concentration).

### Pilot-scale fermentations

Vidal icewine was made in the 2015–2016 winter in Domaine Senpatina IceWine of Huairen in Liaoning Province (Northeast China). Grapes were harvested, destemmed, crushed, and pressed at −8°C to −9°C, and the grape juice (400 g/L of sugar, 13 g/L of total acidity, and a pH of 3.35) was transferred to a 50-L stainless steel fermenter containing 45 L Vidal grape juice with 80 mg/L SO_2_ and 40 mg/L pectinase HC (Lallemand, France) addition. The fermentation temperature was maintained at 16°C. The single fermentations of four *S. cerevisiae* strains and the mixed fermentations of two non-*Saccharomyces* strains with *SC*45 strain were carried out as followed: (1) single inoculation with *SC*42 (*SC*42); (2) single inoculation with *SC*45 (*SC*45); (3) single inoculation with XR (XR); (4) single inoculation with R2 (R2); (5) simultaneous inoculation of *MP*20 and *SC*45 (SI-*MP*20/*SC*45); (6) sequential inoculation of *MP*20 followed by *SC*45 after 2 days (SE-2-*MP*20/*SC*45); (7) sequential inoculation of *MP*20 followed by *SC*45 after 4 days (SE-4-*MP*20/*SC*45); (8) simultaneous inoculation of *HV*11 and *SC*45 (SI-*HV*11/*SC*45); (9) sequential inoculation of *HV11* followed by *SC*45 after 2 days (SE-2-*HV*11/*SC*45); and (10) sequential inoculation of *HV11* followed by *SC*45 after 4 days (SE-4-*HV*11/*SC*45). Total inoculation amount in each treatment was controlled at 1 × 10^6^ viable cells/mL for *S. cerevisiae* and 1 × 10^7^ viable cells/mL for non-*Saccharomyces*. Every experiment was set up in duplicate. Fermentation kinetics and yeast growth were monitored by determining specific gravity and viable cell counts every day. The alcoholic fermentations proceeded for 26 d (pure fermentation) and 30 d (mixed fermentation). After alcoholic fermentation, the final samples were centrifuged to remove yeast cells and stored at −20°C for analysis of residual sugar (glucose and fructose), main products (glycerol, ethanol, acetic acid, and other nonvolatile acids), and volatile compounds.

### Analytical techniques

The quantity of yeasts was determined by plating on a WL nutrient agar containing 100 mg/L chloramphenicol inhibiting bacterial growth. Plates were incubated at 30°C for 72 h. On WL nutrient agar plates, yeast colonies belonging to *S. cerevisiae* and non-*Saccharomyces* were distinguished by their different morphological characteristics. Ethanol, acetic acid, glucose, fructose, glycerol, and organic acids were determined by an HPX-87H Aminex ion-exchange column (300 × 7.8 mm, Bio-Rad Laboratories, Hercules, CA, USA) with 5 mM sulfuric acid as the mobile phase (Duan et al., 2015). The volatile compounds of final wines were determined by headspace solid-phase micro-extraction coupled with gas chromatography–mass spectrometry (HS-SPME-GC-MS) according to our previous study (Zhang et al., 2011; Xu et al., 2015; Liu et al., 2016). Identification of the aroma compounds was based on retention indices of reference standards and mass spectra matching in the standard NIST 11 library. For quantification of these compounds, the relative peak area of each identified compound was measured and then compared with the relative peak area of the added internal standard. Analyses were performed in triplicate. The detailed quantitation information about quantitative ion, quantitative standards, calibration curves, and R^2^ for the quantification of volatile compounds used in this study was provided in Table S2.

### Data analysis

One-way ANOVA using the Duncan test at significance level *p* ≤ 0.05 was carried out to uncover statistical differences between the wines produced from the different inoculation protocols. Principal component analysis (PCA) was carried out using the concentrations of volatile compounds to visualize the differences between wines fermented by different strains and inoculation methods. Statistical analyses were performed with the IBM SPSS Statistical Package (version 24.0, IBM Corp, NY, USA).

## Results

### The cell growth and main fermentation products

**Figure 1:**
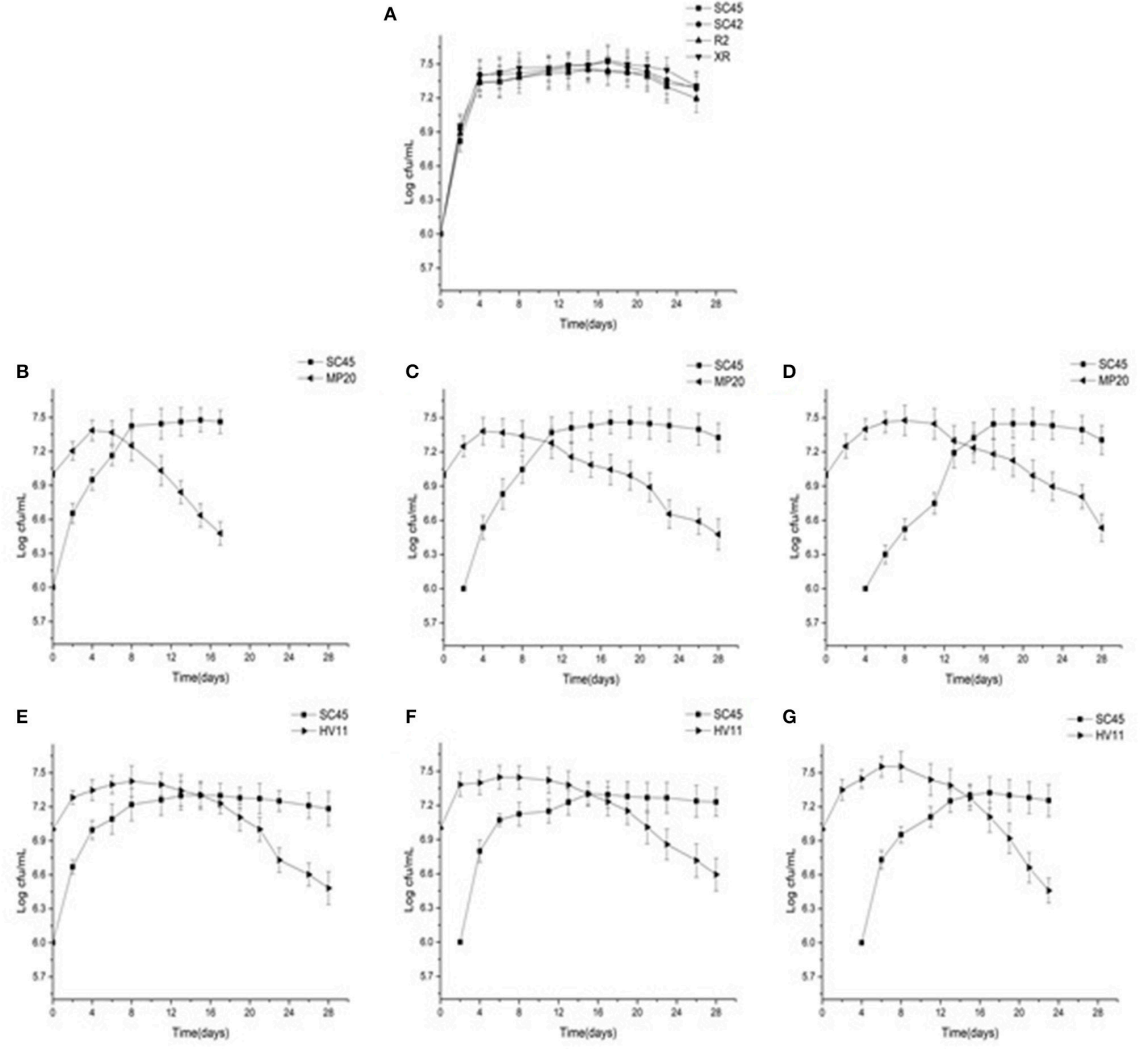
Growth profiles of four *S. cerevisiae* strains in pure fermentation and *S. cerevisiae and* non*-Saccharomyces* strains in mixed fermentations of *MP*20*/SC*45 *and HV*11/*SC*45 during alcoholic fermentation of vidal icewine. **(A)** Four *S. Cerevisiae* strains single fermentation. **(B)** Simulataneous inoculation of *MP*20 and *SC*45 (SI-*MP*20*/SC*45). **(C)** Sequential inoculation of *MP*20 followed by *SC*45 after 2 days (SE-2-*MP*20/*SC*45). **(D)** Sequential inoculation of *MP*20 followed by *SC*45 after 4 days (SE-4-*MP*20/*SC*45). **(E)** Simultaneous inoculation of *HV*11 and *SC*45 (SI-*HV*11/*SC*45). **(F)** Sequential inoculation of HV11 followed by *SC*45 after 2 days (SE-2-*HV*11\*SC*45). **(G)** Sequential inoculation of *HV*11 followed by *SC*45 after 4 days (SE-4-*HV*11/*SC*45).

The growth dynamics of *S. cerevisiae* and non-*Saccharomyces* in pure cultures or mixed cultures are presented in Figure 1. Four *S. cerevisiae* showed similar growth profiles in pure cultures, and reached the maximum cell number of around 3 × 10^7^ CFU/mL after 4 days (Figure 1A). In mixed fermentations, inoculation of non-*Saccharomyces* decreased the cell growth rate of *S. cerevisiae* in comparison with that of pure culture because the time of *SC*45 reaching the highest population (3 × 10^7^ CFU/mL) was delayed by 4–12 days. The strong inhibitions were observed in later inoculation of *S. cerevisiae* (4 days inoculation). *M. pulcherrima* and *H. vineae* reached the maximum population after 4–8 d fermentation. After this, they rapidly decreased although the inoculation amount of *MP*20 and *HV*11 was ten-folds of *SC*45. Simultaneously, *S. cerevisiae* gradually became the dominant strains. The decreased cell number of non-*Saccharomyces* could be associated with their low ethanol tolerance or to the production of other toxic compounds besides ethanol (Tofalo et al., 2016).

The main fermentation products including ethanol, glycerol, acetic acid, nonvolatile acids, and residual sugars are presented in Table 1. The ethanol concentrations were much different in all trials (10.89–12.36%) with the lowest level in SE-2-*MP*20/*SC*45 and the highest level in SI-*MP*20/*SC*45. Pure cultures of four *S. cerevisiae* strains left similar content of sugar (181.2–193.58 g/L). More sugar was consumed by mixed fermentation and the residual sugar concentrations were further decreased to 160.39–189.94 g/L with the lowest amount in SI-*MP*20/*SC*45. This might explain the data that the highest ethanol content was produced in the wine of SI-*MP*20/*SC*45. Glycerol contents ranged from 9.32 g/L (*SC*42) to 10.28 g/L (*SC*45) with no significant differences between various treatments. The amounts of acetic acid in all wines ranged from 1.41 to 1.71 g/L with the lowest level in *SC*42 wine. No significant differences were observed in the amounts of other organic acids including citric acid, malic acid, lactic acid, oxalic acid, and succinic acid in different icewines.

**Table 1:** Physicochemical parameters of icewine samples.

	**XR**	**R2**	***SC*42**	***SC*45**	**SI-*MP*20/*SC*45**	**SE-2-*MP*20/*SC*45**	**SE-4-*MP*20/*SC*45**	**SI-*HV*11/*SC*45**	**SE-2-*HV*11/*SC*45**	**SE-4-*HV*11/*SC*45**
Residual sugar(g/L)	181.2 ± 2.97b	184.12 ± 8.39bc	183.94 ± 9.93bc	193.58 ± 1.87c	160.39 ± 1.4a	189.94 ± 6.82bc	186.25 ± 5.02b	177.23 ± 1.59b	184.85 ± 5.30b	182.03 ± 4.96b
Glycerol(g/L)	9.38 ± 0.54a	9.66 ± 0.05a	9.32 ± 0.34a	10.28 ± 0.16b	10.14 ± 0.15b	9.54 ± 0.08a	9.67 ± 0.11a	9.71 ± 0.04a	10.16 ± 0.04b	10.02 ± 0.13b
Ethanol(%v/v)	11.86 ± 0.23cd	11.25 ± 0.02b	12.10 ± 0.16d	12.11 ± 0.19d	12.36 ± 0.09e	10.89 ± 0.04a	11.87 ± 0.06cd	11.64 ± 0.15c	11.42 ± 0.16bc	11.16 ± 0.76b
Acetic acid(g/L)	1.63 ± 0.03bc	1.68 ± 0.01cd	1.41 ± 0.03a	1.64 ± 0.02bc	1.71 ± 0.02d	1.64 ± 0.01bc	1.65 ± 0.03bc	1.66 ± 0.02bc	1.65 ± 0.02bc	1.66 ± 0.04bc
Oxalic acid(g/L)	0.35 ± 0.01a	0.44 ± 0.03b	0.42 ± 0.02b	0.42 ± 0.01b	0.36 ± 0.02a	0.37 ± 0.01a	0.42 ± 0.02b	0.36 ± 0.03a	0.36 ± 0.03a	0.45 ± 0.02b
Citric acid(g/L)	0.57 ± 0.02a	0.53 ± 0.05a	0.55 ± 0.03a	0.60 ± 0.11a	0.53 ± 0.05a	0.54 ± 0.04a	0.56 ± 0.02a	0.53 ± 0.06a	0.54 ± 0.09a	0.58 ± 0.01a
Malic acid(g/L)	15.85 ± 0.02b	14.14 ± 0.13a	15.84 ± 0.04b	15.85 ± 0.04b	16.29 ± 0.02c	15.9 ± 0.03b	15.88 ± 0.09b	15.85 ± 0.03b	15.79 ± 0.02b	16.55 ± 0.05d
Succinic acid(g/L)	3.44 ± 0.02a	3.59 ± 0.04a	3.55 ± 0.04a	3.45 ± 0.04a	3.53 ± 0.16a	3.46 ± 0.05a	3.59 ± 0.17a	3.47 ± 0.05a	3.52 ± 0.11a	3.43 ± 0.07a
Lactic acid(g/L)	1.23 ± 0.02a	1.28 ± 0.07a	1.25 ± 0.03a	1.31 ± 0.18a	1.23 ± 0.02a	1.04 ± 0.22a	1.27 ± 0.06a	1.19 ± 0.04a	1.24 ± 0.03a	1.25 ± 0.05a

*XR, S. cerevisiae XR pure fermentation; R2, S. cerevisiae R2 pure fermentation; SC42, S. cerevisiae SC42 pure fermentation; SC45, S. cerevisiae SC45 pure fermentation; SI-MP20/SC45, Simultaneous inoculation of MP20 and SC45; SE-2-MP20/SC45, Sequential inoculation of MP20 followed by SC45 after 2 days; SE-4-MP20/SC45, Sequential inoculation of MP20 followed by SC45 after 4 days; SI- HV11/SC45, Simultaneous inoculation of HV11 and SC45; SE-2- HV11/SC45, Sequential inoculation of HV11 followed by SC45 after 2 days; SE-4- HV11/SC45, Sequential inoculation of HV11 followed by SC45 after 4 days. Values are given as mean ± standard deviation of two biological replicates and three HPLC detection runs. Data with different letters (a, b, c, d, e) within each column are different according to Duncan tests (0.05%)*.

### Volatile aroma compounds in vidal icewine

A total of fifty-two volatiles were detected in all samples (Table S3).The characteristic aroma compounds of Vidal icewine were presented in Table 2, including ten high alcohols, ten esters, four terpenes, one aldehyde, and one C13-norisoprenoid, in which sixteen whose odor activity value (OAV) exceeding one were underlined. It should be mentioned that there were not many references, which have given the thresholds for these aroma compounds in the icewine matrix. Thresholds of Some compounds were, therefore, obtained either from the system of table wine or ethanol solution (Table S2).

**Table 2:** Twenty-six main volatile aroma compounds (μg/L) in icewines obtained with four *S. cerevisiae* pure fermentations and mixed fermentations of non*-Saccharomyces* and *SC*45 after alcohol fermentation.

**Compounds**	**XR**	**R2**	***SC*42**	***SC*45**	**SI-*MP*20/*SC*45**	**SE-2-*MP*20/*SC*45**	**SE-4-*MP*20/*SC*45**	**SI-*HV*11/*SC*45**	**SE-2-*HV*11/*SC*45**	**SE-4-*HV*11/*SC*45**
**C6 ALCOHOL**										
1-Hexanol	1163.1 ± 29.0d	1103.5 ± 1.2bc	1089.2 ± 29.7b	1095.6 ± 1.3bc	1043.4 ± 8.2a	1026.4 ± 19.9a	1036.7 ± 4.3a	1128.2 ± 7.0c	1080.4 ± 5.4b	1172.6 ± 10.4d
(Z)-3-Hexen-1-ol	166.3 ± 0.6a	153.4 ± 10.1a	167.8 ± 7.5ab	154.2 ± 6.9a	163.9 ± 17.1a	171.9 ± 1.4ab	174.8 ± 9.3ab	177.8 ± 2.4ab	186.8 ± 10.8ab	201.4 ± 35.2b
(E)-3-Hexen-1-ol	183.5 ± 5.6a	175.7 ± 23.3a	190.5 ± 23.3a	170.6 ± 2.5a	174.1 ± 15.5a	173.0 ± 7.7a	175.7 ± 0.5a	184.0 ± 7.4a	197.9 ± 38.3a	237.7 ± 8.7b
Total of C6 alcohols	1512.9 ± 24.1e	1432.6 ± 32.2bc	1447.5 ± 60.54bc	1420.4 ± 10.7b	1381.5 ± 40.8a	1371.3 ± 13.6a	1387.3 ± 14.2a	1490.0 ± 16.8d	1465.1 ± 43.7cd	1611.8 ± 54.4f
**HIGHER ALCOHOLS**										
Isobutanol	68448.6 ± 7.1g	67829.7 ± 11.6f	59640.7 ± 38.6b	55943.8 ± 9.0a	87571.6 ± 10.8j	62209.2 ± 0.6c	66621.1 ± 30.4e	83820.9 ± 17.4i	64665.8 ± 39.1d	71131.8 ± 18.3h
Isopentanol	48084.5 ± 42.5i	42758.8 ± 5.1e	40830.5 ± 49.1a	41663.3 ± 8.4c	43591.3 ± 4.9f	45394.8 ± 24.9h	44436.6 ± 3.4g	50179.0 ± 7.9j	41003.3 ± 2.0b	41961.5 ± 0.6d
1-Octen-3-ol	164.5 ± 4.4a	159.0 ± 11.5a	160.9 ± 0.3a	158.4 ± 4.6a	160.8 ± 11.7a	167.8 ± 4.1a	172.2 ± 15.4a	172.0 ± 1.6a	168.2 ± 18.1a	179.7 ± 22.8a
2-Phenylethanol	4008.3 ± 11.5d	2313.3 ± 0.7a	4520.4 ± 38.3f	3195.8 ± 12.4b	4113.9 ± 5.8e	5001.3 ± 1.3g	3519.0 ± 1.7c	7860.6 ± 14.0h	9816.9 ± 2.4i	12229.8 ± 13.9j
2,3-Butanediol	111000.2 ± 10.3e	104000.3 ± 9.55a	104950.0 ± 4.3b	129531.1 ± 5.2j	105872.2 ± 5.8d	113000.7 ± 17.3g	105342.2 ± 13.1c	112430.5 ± 9.9f	113789.7 ± 4.1h	113998.6 ± 9.9i
1-Octanol	10.5 ± 1.3a	30.7 ± 1.1e	27.2 ± 0.1e	31.0 ± 6.7e	30.4 ± 3.4e	26.5 ± 1.8de	26.5 ± 2.0de	19.3 ± 2.5bc	15.3 ± 0.4ab	12.5 ± 4.8ab
2-Octanol	16.4 ± 1.4ab	14.8 ± 1.3ab	15.8 ± 0.1ab	14.8 ± 0.7ab	13.8 ± 1.3ab	12.9 ± 0.4a	12.9 ± 1.8a	16 ± 0.6ab	15.4 ± 2.5ab	17.9 ± 3.5b
Total of higher alcohols	231861.8 ± 87.1c	217197.5 ± 31.7a	210285.0 ± 78.0a	230660.4 ± 17.4c	241459.5 ± 9.3d	225960.7 ± 0.7b	220257.5 ± 18.1b	254706.1 ± 9.1e	229671.1 ± 33.6bc	239811.8 ± 58.5d
**ACETATE ESTERS**										
Ethyl acetate	7045.6 ± 30.8c	7871.2 ± 0.1h	7441.5 ± 9.7e	5626.4 ± 8.0a	8762.7 ± 13.3j	7147.5 ± 43.7d	7556.6 ± 40.5f	8532.3 ± 7.4i	6946.4 ± 1.0b	7785.7 ± 8.6g
2-Phenethyl acetate	3103.6 ± 42.7e	2017.5 ± 10.7a	2774.7 ± 21.1c	2590.8 ± 11.4b	2878.8 ± 25.8d	3446.0 ± 35.1g	3197.4 ± 47.7f	4556.4 ± 41.9h	8341.6 ± 0.1j	6716.0 ± 15.4i
Isoamyl acetate	1209.0 ± 0.9f	863.3 ± 2.5a	993.0 ± 1.9bc	963.6 ± 27.9b	1031.0 ± 10.4cd	1075.9 ± 3.2d	1062.6 ± 2.2d	1129.1 ± 31.3e	1048.0 ± 41.4d	1047.0 ± 2.0d
Hexyl acetate	169.5 ± 0.7de	121.5 ± 0.5cd	140.6 ± 14.8ab	129.2 ± 0.2bc	81.9 ± 1.8a	158.5 ± 3.1e	148.0 ± 3.8f	142.2 ± 2.4c	72.5 ± 5.4e	126.7 ± 3.7f
Total of acetate esters	11608.5 ± 4.1c	10944.2 ± 17.5b	11426.0 ± 27.8c	9385.3 ± 5.7a	12996.4 ± 1.8d	11909.3 ± 1.5c	12048.1 ± 85.1d	14441.5 ± 0.9e	16485.6 ± 50.8g	15755.1 ± 10.8f
**ETHYL ESTERS**										
Ethyl butanoate	264.4 ± 40.0bc	224.3 ± 37.7ab	302.1 ± 1.5c	233.4 ± 26.7ab	316.1 ± 5.8c	246.2 ± 5.2ab	213.9 ± 23.9ab	249.0 ± 10.9ab	199.6 ± 11.3a	233.9 ± 20.3ab
Ethyl hexanoate	2543.8 ± 32.9d	2261.9 ± 13.9b	2361.6 ± 26.4a	3119.9 ± 35.1f	2814.4 ± 6.4e	2136.8 ± 8.5a	2249.8 ± 6.1b	2240.4 ± 24.5b	2844.8 ± 10.9e	2569.9 ± 40.1d
Ethyl octanoate	574.1 ± 5.6d	482.9 ± 1.0c	681.7 ± 19.7f	1909.9 ± 2.8i	292.5 ± 4.8a	867.4 ± 4.5h	566.9 ± 9.2d	720.5 ± 12.4g	621.4 ± 11.2e	420.1 ± 7.2b
Ethyl decanoate	523.5 ± 21.7b	542.1 ± 7.5b	604.7 ± 49.8c	719.0 ± 16.4e	479.9 ± 20.1a	795.7 ± 2.5f	656.9 ± 9.0d	608.9 ± 1.8c	548.5 ± 2.9b	680.9 ± 5.2de
Ethyl lactate	1907.1 ± 36.3e	1361.3 ± 19.7b	1577.5 ± 24.8c	1068.9 ± 18.1a	1538.2 ± 44.7c	1349.4 ± 6.4b	1526.5 ± 2.0c	1762 ± 18.1d	1770.3 ± 3.1d	1717.2 ± 17.0d
Ethyl phenylacetate	40.7 ± 0.0ab	40.7 ± 0.0ab	40.7 ± 0.0ab	40.4 ± 0.3a	43.8 ± 4.1b	40.9 ± 0.1ab	40.8 ± 0.1ab	41.0 ± 0.2ab	41.4 ± 0.3ab	41.3 ± 0.0ab
Total of ethyl esters	5957.7 ± 74.6cd	5016.3 ± 49.1a	5677.5 ± 20.1bc	7212.9 ± 60.4e	5574.0 ± 33.3b	5565.4 ± 3.0b	5384.3 ± 17.5ab	5747.6 ± 15.5c	6164.5 ± 30.2d	5786.8 ± 31.9c
Total of all esters	19088.9 ± 62.9d	17520.5 ± 59.7a	18654.2 ± 37.2c	18170.6 ± 37.6b	20130.3 ± 29.1e	19002.4 ± 10.8d	18981.6 ± 71.4d	21702.5 ± 6.4f	24146.5 ± 83.4h	23030.2 ± 46.0g
**TERPENES**										
Linalool	42.4 ± 0.2ab	41.5 ± 0.5ab	40.9 ± 0.4a	42.0 ± 0.9ab	41.6 ± 1.3ab	43.5 ± 0.0bc	44.6 ± 0.7cd	45.4 ± 0.7cde	46.6 ± 0.2de	47.2 ± 2.3e
Geraniol	1924.0 ± 1.2ab	1921.7 ± 0.1a	1924.4 ± 0.2ab	1936.4 ± 17.5b	1921.1 ± 1.0a	1925.7 ± 0.8ab	1924.8 ± 1.5ab	1926.3 ± 2.9ab	1930.5 ± 1.5ab	1931.3 ± 0.1ab
*cis*-Rose oxide	14.9 ± 0.9d	9.9 ± 1.8b	7.9 ± 0.4a	14.0 ± 5.1c	16 ± 1.5g	15.3 ± 0.2f	15.1 ± 1.5e	16.7 ± 0.4h	17.2 ± 2.9i	18.2 ± 1.3j
β-Citronellol	20.3 ± 0.2ab	19.2 ± 0.3ab	21.2 ± 0.0abc	20.2 ± 2.7ab	17.8 ± 0.2a	23.1 ± 0.6bc	22.0 ± 0.6abc	22.0 ± 1.7abc	25.6 ± 4.4c	25.4 ± 1.0c
Total of terpenes	2013.3 ± 4.7abc	2004.3 ± 1.3a	2009.2 ± 2.0ab	2050.3 ± 43.2c	2007.0 ± 5.4ab	2025.1 ± 5.8abc	2021.5 ± 9.4abc	2029.2 ± 7.0abc	2043.2 ± 3.5abc	2044.9 ± 4.2bc
**OTHERS**										
β-Damascenone	42.3 ± 5.1b	41.9 ± 0.9b	40.0 ± 3.6a	48.1 ± 15.5c	40.9 ± 9.5ab	55.6 ± 0.6d	62.7 ± 3.9e	67.1 ± 10.8f	101.7 ± 37.1h	96.5 ± 21.0g
Phenylacetaldehyde	1.6 ± 1.5ab	2.5 ± 0.7ab	1.4 ± 1.6ab	4.5 ± 2.4ab	0.3 ± 0.1a	2.8 ± 1.8ab	4.6 ± 0.8ab	3.2 ± 0.7ab	10.3 ± 9.6b	5.1 ± 5.8ab

*XR, S. cerevisiae XR pure fermentation; R2, S. cerevisiae R2 pure fermentation; SC42, S. cerevisiae SC42 pure fermentation; SC45, S. cerevisiae SC45 pure fermentation; SI-MP20/SC45, Simultaneous inoculation of MP20 and SC45; SE-2-MP20/SC45, Sequential inoculation of MP20 followed by SC45 after 2 days; SE-4-MP20/SC45, Sequential inoculation of MP20 followed by SC45 after 4 days; SI- HV11/SC45, Simultaneous inoculation of HV11 and SC45; SE-2- HV11/SC45, Sequential inoculation of HV11 followed by SC45 after 2 days; SE-4- HV11/SC45, Sequential inoculation of HV11 followed by SC45 after 4 days. Sixteen odor active compounds (OVA > 1) were underlined. Values are given as mean ± standard deviation of two biological replicates and three HPLC detection runs. Data with different letters (a, b, c, d, e, f, g, h, i, j) within each column are different according to Duncan tests (0.05%)*.

### C_6_ alcohols and other higher alcohols

The C6 alcohols including 1-hexanol, (Z)-3-hexen-1-ol, and (E)-3-hexen-1-ol usually have the character of “vegetal” and “herbaceous” note and cause a negative effect on wine aroma (Ferreira et al., 2000). The XR and SC45 produced the highest (1512.9 μg/L) and lowest (1420.4 μg/L) total levels of C_6_ alcohols in pure cultures, respectively (*p* < 0.05). Coinoculation of *H. vineae* with SC45 further increased C_6_ alcohols compared with *SC*45 monoculture and the highest contents (1611.8 μg/L) were found in SE-4-*HV*11/*SC*45, followed by SI-*HV*11/*SC*45, and SE-2-*HV*11/*SC*45. In comparison, *MP*20/*SC*45 produced less C_6_ alcohols than *SC*45 monoculture. Seventeen higher alcohols were identified in this study (Table S3), and five compounds (isobutanol, isopentanol, 1-octen-3-ol, 2, 3-butanediol, and 2-phenylethanol) exceed their odor threshold. *SC*42 and SI-*HV*11/*SC*45 produced the lowest (210.3 mg/L) and the highest contents (254.7 mg/L), respectively (*p* < 0.05). Among *S. cerevisiae* strains, XR is a strong producer of higher alcohol due to generating more isobutanol, isopentanol, and 1-octen-3-ol. *SC*42 produced the highest level of 2-phenylethanol (4520.4 μg/L) among four *S. cerevisiae* strains. 2-Phenylethanol (floral note) is a desired aroma compound in wines (Mendes et al., 2012). *SC*45 formed comparable amount of higher alcohol with XR. Interestingly, co-inoculation of *SC*45 with *HV*11 and *MP20* significantly improved the production of higher alcohols (including isobutanol, isopentanol, isopentanol, 2-phenylethanol, and total content). The SI *MP*20/*SC*45 produced the highest concentration of isobutanol, followed by SI *HV*11/*SC*45, which was 56.5 and 49.8% higher than that of *SC*45 monoculture (*p* < 0.05), and also 27.9 and 22.4% higher than that of XR monoculture (*p* < 0.05). Isopentanol content in SI-*HV*11/*SC*45 wine was increased by 20.4% compared to *SC*45 wine (*p* < 0.05). More importantly, SE-4-*HV*11/*SC*45 produced the highest amount of 2-phenylethanol, which was 2.82, 2.05, and 1.70 folds higher than those of monocultures of *SC*45, XR, and *SC*42, respectively.

### Esters

Eighteen esters were identified in all samples (Table S3) and seven compounds exceeded the individual odor threshold in this study, including three acetate esters (ethyl acetate, 2-phenethyl acetate, isoamyl acetate) and four ethyl esters (ethyl butanoate, ethyl hexanoate, ethyl octanoate, ethyl decanoate) (Table 2). The highest and lowest total concentrations of esters were produced by SE-2-*HV*11/*SC*45 (24146.5 μg/L) and R2 monoculture (17520.5 μg/L), respectively (*p* < 0.05). Different *S. cerevisiae* strains had distinct impact on formation of particular esters. The XR produced the maximum concentration of isoamyl acetate (1209.0 μg/L), hexyl acetate (169.5 μg/L), and ethyl lactate (1907.1 μg/L). R2 generated more ethyl acetate, but lower contents of ethyl octanoate, 2-phenethyl acetate, and isoamyl acetate. Indigenous *SC*42 was characterized by the highest amount of ethyl butanoate, while *SC*45 was featured by the highest amount of ethyl hexanoate, ethyl octanoate, and ethyl decanoate among four *S. cerevisiae* strains; especially, for ethyl octanoate (1909.9 μg/L), the value was 2.95 and 2.33 folds higher than those of R2 and XR strains, respectively. Similar to higher alcohols, mixed fermentations led to different profiles of esters formation. Mixed fermentations promoted the productions of acetate ester, especially *HV*11/*SC*45. The highest amount of acetate esters was formed by SE-2-*HV*11/*SC*45, and 2-phenethyl acetate (fruity and floral note) was 2.22 and 1.69 folds higher than those of *SC*45 and XR, respectively. Conversely, mixed fermentations decreased most ethyl esters (ethyl hexanoate, ethyl octanoate, and ethyl decanoate) relative to *SC*45 monoculture with the exception of ethyl lactate, and the pronounced decrease was observed in the wine of SE-4-*MP*20/*SC*45. Although resulting in decrease of fatty acid ethyl esters, the high increase of acetate esters led to the highest contents of esters still achieved in SE-2-*HV*11/*SC*45 wine (24146.5 μg/L), which was a 32.9% increaes relative to *SC*45 wine (*p* < 0.05).

### Terpenes, C13-norisoprenoid, and aldehyde

Terpenes and C13-norisoprenoids are derived from grapes and have a major positive impact on floral aroma of wines directly or through synergistic effects (Swiegers and Pretorius, 2005). Six terpenes were detected in all samples and three compounds with OAV above one, including linalool (sweet and floral note), geraniol (floral note), and *cis*-rose oxide (floral note). There were no significant differences in the contents of linalool (40.9–47.2 μg/L) and geraniol (1921.1–1936.4 μg/L) among all samples, while *cis*-rose oxide, generating lychee and rose smell to Vidal icewine (Ma et al., 2017), showed significant increment by mixed fermentation with 30.0% raise in SE-4-*HV*11/*SC*45 wine relative to *SC*45 wine (*p* < 0.05), followed by SE-2-*HV*11/*SC*45 wine and SI *HV*11/*SC*45 wine. β-Damascenone and phenylacetaldehyde are the key odorants in Vidal icewine and impart floral and honey notes to icewine (Ma et al., 2017). Due to low sensory threshold (0.05 and 1 μg/L, respectively), small variation of their concentrations can result in significant influence on the entire aroma profiles of wines. Both compounds exceeded individual sensory threshold in all samples, and the highest values were found in the *SC*45 wine among the four monoculture wines. As expected, *HV*11/*SC*45 further increased the concentrations of both compounds. The content of β-damascenone in SE-2-*HV*11/*SC*45 wine was 111.4% higher than that of *SC*45 wine (*p* < 0.05), and phenylacetaldehyde concentration achieved 128.9% increment although having no statistical difference between two treatments (*p* < 0.05).

### The PCA profiles and aroma series mode

**Figure 2:**
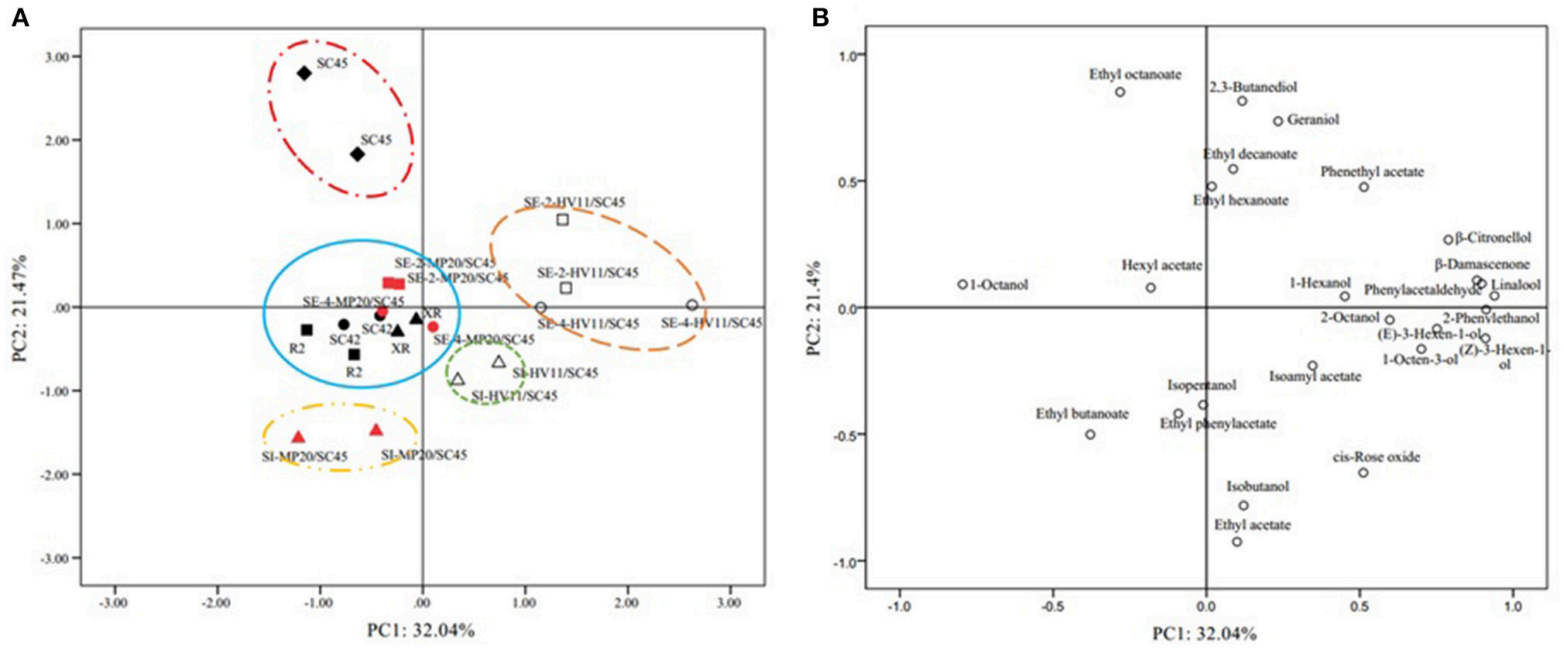
Principal Component Analysis (PCA) biplots of icewine products resulting from twenty-five odor active compounds (OVA > 0.1) produced by ten treatments used different strains and inoculation methods in 50 L fermenters. Score plot **(A)** and loading plot **(B)**. The data are from duplicate biological repeats with three technical replicates each.

The above data indicated that cofermentation of *S. cerevisiae* with *M. pulcherrima* or *H. vineae* could generate diversified volatile profiles of wines. To highlight the differences of fermentation by different strains and inoculation methods and to identify the volatile compounds that discriminate these treatments, PCA was applied using twenty-five aromatic compounds where OAV exceeded 0.1. As shown in Figure 2A, the first and second accounted for 38.3% (PC1) and 21.8% (PC2) of the total variation, respectively. The PCs roughly distinguished wine samples fermented by different inoculations. Wines produced by *HV*11/*SC*45, especially sequential inoculation, were clearly separated by PC1 from the other wines, suggesting that *HV*11/*SC*45 had a higher potential to generate distinct aromatic profile than that of *MP*20/*SC*45. The main responsible components for this separation were β-citronellol, β-damascenone, linalool, *cis*-rose oxide, phenylacetaldehyde, 2-phenylethanol, 2-phenethyl acetate, (Z)-3-hexen-1-ol, and 1-octen-3-ol (Figure 2B). The PC2 could discriminate the wine of *SC*45 from other wines of monocultures mainly by ethyl octanoate. These data indicated that the complexity of the aromatic property of *S. cerevisiae* can be further increased by cofermentation with *H. vineae*. In comparison, *MP*20/*SC*45 had less ability to diversify the aromatic profiles although the wine made by SI *MP*20/*SC*45 was separated from *SC*45 wine by producing more ethyl butanoate.

**Figure 3:**
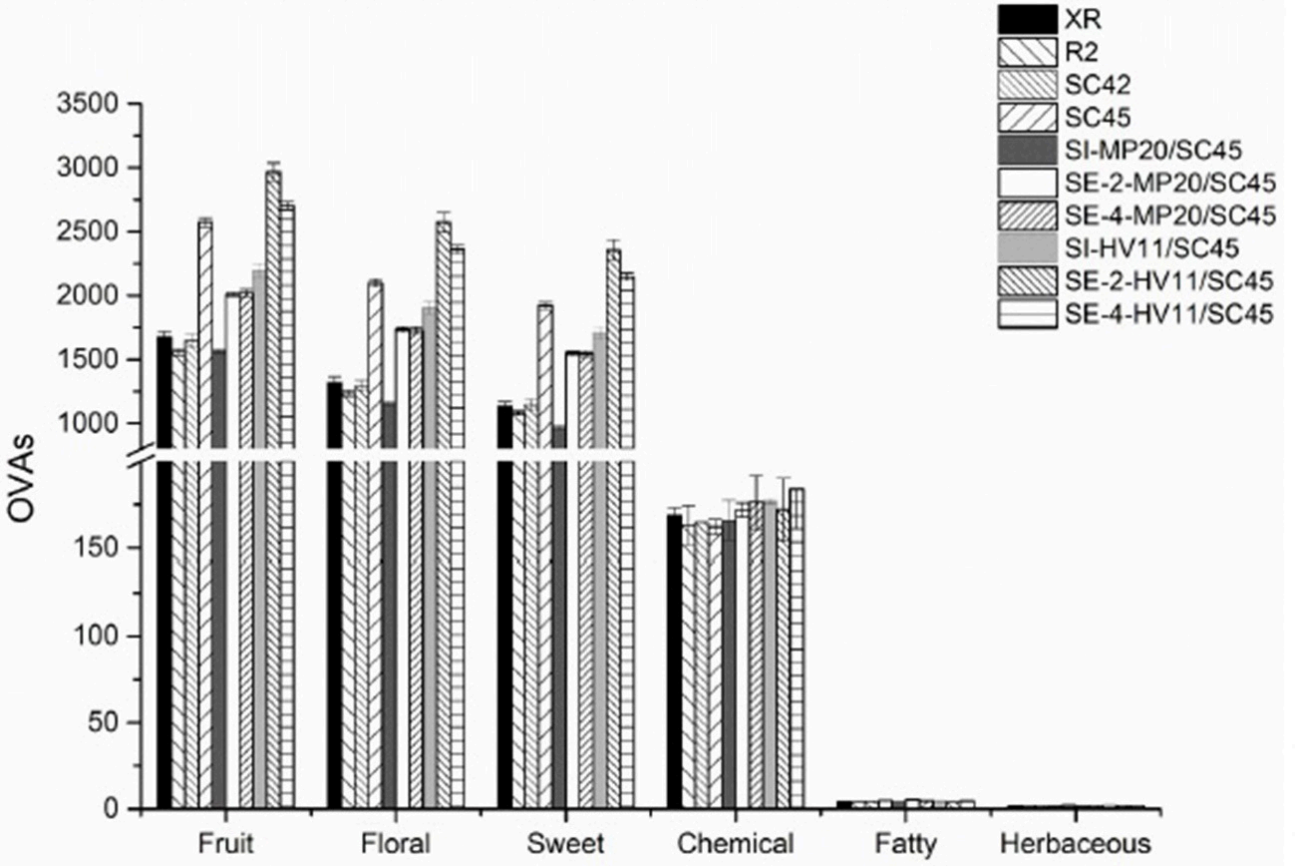
Aroma series in final icewines produced by four *S.cerevisiae* strains single fermentation and the mixed fermentations of *MP*20/*SC*45 and *HV*11/*SC*45, respectively. Error bars represent standard deviations. Aroma compounds (OVA > 1) are calculated for fruit series: ethyl acetate, ethyl butanoate, ethyl hexanoate, ethyl octanote, ethyl decanoate, 2-phenethyl acetate, isoamyl acetate, and β-damascenone; for floral series: 2-phenethyl acetate, ethyl octanoate, 2-phenylethanol, phenylacetaldehyde, β-damascenone, linalool, geraniol and *cis*-rose oxide; for sweet series; ethyl octanoate, 2-phenylethanol, phenylacetaldehyde, linalool, and β-damascenone; for chemical series: ethyl acetate, isobutanol, isopentanol, and 1-octen-3-ol; for fatty series: ethyl decanoate and isopentanol; for herbaceous series: isobutanol.

To better understand the influence of cofermentations with selected non-*Saccharomyces* strains on the icewine odor profile and the contributions of various aroma compounds to the olfactory impression of icewine, an aromatic series was further established by the combination of OAVs of a group of volatile active compounds with similar odor descriptions (Peinado et al., 2006; Wu et al., 2011). In this research, twenty-five aroma compounds showed an OAV above 0.1 (Table 2). According to previous researches (Buettner et al., 2003; Peinado et al., 2006; Yang et al., 2008; Zhang et al., 2011), these compounds mainly associated with the aroma attributes of “alcohol,” “solvent,” “green,” “bitter,” “fatty,” “solvent,” “nail polish,” “rancid,” “mushroom,” “rose,” “honey,” “fruity,” “butter,” “sweet,” “jasmine,” “lemon,” “pineapple,” “balsamic,” “banana,” “strawberry,” “pear,” “cherry,” “geranium,” and “lychee” (Table S2). Six aromatic series of volatile compounds were, therefore, established, including fruity, floral, sweet, chemical, fatty, and herbaceous (Figure 3). Of these, the fruity, floral, and sweet series were prominent, followed by the chemical, fatty, and herbaceous series. Wines of *SC*45 monoculture had much higher OAVs of fruity, floral, and sweety among *S. cerevisiae* strains due to production of higher amounts of ethyl octanoate, ethyl decanoate, β-damascenone, and phenylacetaldehyde. In comparison, chemical, fatty, and herbaceous aroma series were equal in monoculture wines. As regarding to mixed fermentation, *MP*20/*SC*45 produced relatively higher values of fruity, floral, and sweety aroma series than those of XR, R2, and *SC*42, but was inferior to *SC*45 monoculture. This suggested that combination of *MP*20 and *SC*45 might be not an effective pair to enhance the aromatic quality of icewine. Conversely, sequential inoculation of *SC*45 and *HV*11 significantly increased the features of fruity, floral, and sweety compared to *SC*45 monoculture, especially SE-2*-HV*11/*SC*45, where the values of fruity, floral, sweety series were 15.3, 22.5, and 22.6% higher than those of *SC*45 monoculture. The increments of higher alcohols (isobutanol, isopentanol, and 2-phenylethanol), acetate esters (2-phenethyl acetate and isoamyl acetate), *cis*-rose oxide, β-damascenone, and phenylacetaldehyde were mainly responsible for these improvements.

## Discussion

Discovery of *Saccharomyces* and non-*Saccharomyces* strains with suitable inoculation methods is an effective method to produce wine products with desired and diversified aroma characteristics as expected by consumers. The aim of this study was to evaluate whether the multiculture of indigenous *M. pulcherrima MP*20 or *H. vineae HV*11 strains with *S. cerevisiae* could also produce Vidal icewine with diversified aroma quality, respectively. The obtained results showed that *S. cerevisiae* strains, paired strains of non-*Saccharomyces*, and inoculated methods had distinct effects on the formation of aromatic compounds in final icewines.

The contribution of wine yeast to the final aromatic characteristics of wine is largely dependent on the persistence of strains and cell number during alcoholic fermentation. At present, there are few wine yeast strains available for icewine fermentation. The non-*Saccharomyces* strains *MP*20 and *HV*11 used in this work were isolated from spontaneous fermentation of Vidal icewine. The preliminary experimental results showed that *MP*20 and *HV*11 strains have good technological characteristics, including high tolerance to ethanol, SO_2_, and higher sugar, low production of H_2_S, and high production of protease and glucosidase (Table S1). The results of fermentation trials also showed that both non-*Saccharomyces* strains can well multiply and persist in initial stages of icewine fermentation, although they were gradually dominated by *S. cerevisiae*. These data well verified the feasibility of selecting suitable non-*Saccharomyces* strains from spontaneous fermentation for wine production (Regodón et al., 1997; De Benedictis et al., 2011). Actually, the strategy of imparting a particular stress factor in spontaneous wine fermentation for the purpose of isolating yeast strains with desired physiological characteristics has been successfully applied by different researchers. Stress factors include high tolerance to low temperature (Massera et al., 2012), reducing copper content in wine (Capece et al., 2017), and fermenting very high-gravity worts (Huuskonen et al., 2010). Our data showed that *HV*11 had better growth profile than that of *MP*20 and a higher growth rate and cell number were observed during fermentation, which was consistent with previous results that *H. vineae* has a higher ability to adapt to harsh conditions of wine fermentation compared to other non-*Saccharomyces* species (Viana et al., 2011). There is one concern wherein the initial growth of apiculate yeast may be inhibitory for subsequent *S. cerevisiae* growth and result in sluggish fermentations (Medina et al., 2013). The inhibition on the growth of *S. cerevisiae* (*SC*45) by inoculating *HV*11was not observed in this study, implying that *HV*11 has great potential in industrial icewine production.

Icewine juice is a concentrated mix of all juice components such as sugars and acids. To combat the osmotic stress imposed by the icewine juice, yeast cells may allocate carbon resources derived from sugar metabolism toward the production of metabolites necessary for adaptation and survival such as acetic acid and glycerol (Heit et al., 2018). The upper allowable limit of acetic acid is 2.1 g/L in Canadian (Pigeau and Inglis, 2005) and Chinese (GB/T25504-2010, 2010^2^) icewines. The amounts of acetic acid present in all samples were below this value (range in 1.41 to 1.71 g/L). Glycerol is one important product of yeast fermentation and is typically found at concentrations of 4-10 g/L in dry wine. In general, higher glycerol levels are considered to improve wine quality. In this study, the highest content of glycerol (10.28 g/L) was produced in *SC*45 wine, which was not consistent with the previous literature wherein sequential inoculation of *H. vineae* and *S. cerevisiae* could result in significant increase of glycerol content in Chardonnay wine (Medina et al., 2013). *M. pulcherrima* is one commercial non-*Saccharomyces* and usually used to reduce wine alcohol. The lowest and highest levels of alcohol were achieved in SE-2-*MP*20/*SC*45 and SI-*MP*20/*SC*45, respectively, which well confirmed that the inoculation method is essential for cofermentation of *M. pulcherrima* and *S. cerevisiae* to reduce ethanol content of wine (Varela et al., 2016).

Consistent with previous literature (Erasmus et al., 2004; Crandles et al., 2015), we found that different inoculated *S. cerevisiae* strains produced distinct aromatic profiles of icewine products. Compared to R2, the commercial XR strain produced the highest levels of higher alcohols and acetate esters, revealing its good enological properties in icewine production. Esters (acetate esters and fatty acid ethyl esters) are the major volatile constituents in wines (Sumby et al., 2010). Ethyl hexanoate, ethyl isobutanoate, ethyl 2-methyl-butanoate, ethyl isovalerate, and ethyl butanoate are major esters in Vidal icewine and provide desired fruit flavor to icewine (Ma et al., 2017). Indigenous yeast *SC*45 formed higher amounts of ethyl esters (ethyl octanoate, ethyl hexanoate, and ethyl decanoate). As regarding to mixed fermentation, *HV*11/*SC*45 further improved the production of volatiles relative to *SC*45 monoculture, but this production was largely dependent on inoculated method. SI-*HV*11/*SC*45 produced the highest concentration of C_6_ alcohols and higher alcohols, while SE-2*-HV*11/*SC*45 and SE-4*-HV*11/*SC*45 generated more 2-phenylethanol, acetate esters (2-phenethyl acetate), *cis*-rose oxide, β-damascenone, and phenylacetaldehyde. These results were consistent with previous literatures wherein there was the promotion of *H. vineae* and *S. cerevisiae* for 2-phenylethyl acetate formation (Viana et al., 2011; Medina et al., 2013). Compared to *H. vineae, MP*20/*SC*45 has less ability to reshape the aromatic profile of icewine. Inoculation of *M. pulcherrima* could improve ethyl acetate, 2-methyl propanol, and 2, 3-methyl butanol in table wine (Varela et al., 2017). These desired enological traits were not observed in this work. The inconsistent data could be due to the differences of strains and (or) composition of grape must. Thus, it is essential to comprehensively evaluate the aromatic property of one particular strain before using it to produce different types of wines.

It should be noticed that *HV*11/*SC*45 and *MP*20/*SC*45 led to decreased production of fatty acid ethyl esters compared to the monoculture of *SC*45, which corresponded to the previous data that *H. vineae* produced lesser amounts of ethyl esters including ethyl butyrate, ethyl hexanoate, and ethyl octanoate than that of *S. cerevisiae* (QA23) (Lleixà et al., 2016). At present, we are not able to explain these phenomena well enough. Considering that the *SC*45 is the strongest strain to produce fatty acid ethyl esters, we assumed that initial inoculation of non-*Saccharomyces* strains *(M. pulcherrima* and *H. vineae)* with lower ability to form ethyl esters negatively influenced *Saccharomyces* growth and formation of ethyl esters by *SC*45. This assumption could be partially supported by the observation that the later inoculation of *SC*45 induced greater decrease of ethyl esters. The detailed mechanisms need to be further explored at metabolites and transcriptional levels.

*Hanseniaspora* species are usually regarded as spoilage yeast in wine fermentation. Using *H. vineae* as the starter has recently received great interest because it produces several key aromatic compounds and increases flavor diversity (Lleixà et al., 2016). However, sensory evaluation of wines produced by this apiculate yeast in laboratory studies is still limited and the results have not been consistent. For example, five and ten folds higher levels of 2-phenylethyl acetate ester were produced in wine of *H. vineae* cofermentation than conventional and spontaneous fermentations, respectively, but 2-phenylethanol was significantly lower than those of the other two treatments (Medina et al., 2013). High production of 2-phenylethyl acetate by *H. vineae* was also confirmed previously in laboratory fermentations (Viana et al., 2009, 2011). Our results were in agreement with the previous literature. In addition to 2-phenylethyl acetate, other desired compounds such as 2-phenylethanol, β-damascenone, isoamyl acetate, and phenylacetaldehyde were found simultaneously increased by *HV*11/*SC*45. Recently, Huang et al. (2018) compared the flavor characteristics of Vidal icewines from China and Canada and established relationships between their sensory descriptors and chemical composition. Their results indicated that Vidal icewines produced in Huairen were mainly characterized by isoamyl acetate, 2-phenylethyl acetate, and 2-phenylethanol, and expressed nut and honey aromas. *HV*11 used in this study was isolated from the Huairen region. We, therefore, inferred that *H. vineae* is a unique non-*Saccharomyces* species in Huairen and imparts the Vidal icewine with typical sensory characteristics. This deduction deserves to be further investigated. We also found that the formations of volatiles by multicultures were largely affected by the timing of inoculation of *S. cerevisiae* strain. The later inoculation of *S. cerevisiae* is favorable for most volatile formations, which was more pronounced in sequential fermentation of *HV*11 and *SC*45.

In this work, we established the aromatic series to evaluate the effects of multicultures on aromatic quality of icewine. This method is often used to determine the contribution of different aroma compounds to olfactory impression of wine and achieve the information of volatile variation on aroma qualities of wines (Peinado et al., 2006; Wu et al., 2011). The results showed that SE-2*-HV*11/*SC*45 obtained the highest values of fruity, floral, and sweety series among all treatments, which was corresponding to the high levels of 2-phenylethanol, 2-phenethyl acetate, isoamyl acetate, *cis*-rose oxide, β-damascenone, and phenylacetaldehyde produced in this wine. Considering the positive contribution of these volatiles to the aroma quality of the wine, the improvement of aromatic quality by *HV*11/*SC*45 can be expected. Certainly, to further confirm this conclusion, sensory evaluation needs to be conducted. In summary, the present data verified the positive enological characteristics of non-*Saccharomyces* strains (*H. vineae* and *M. pulcherrima*) in icewine fermentation. Especially, the sequential inoculation of *H. vineae* with *S. cerevisiae* has a significant impact on aromatic quality, and efficiently improved the aromatic diversity of icewine products. To further verify this conclusion, more work with *HV11* strains needs to be done along with other grape varieties such as Riesling and also in larger scale fermenters. The relevant experiments will be done in our future work.

## Conclusion

The present results indicated that multicultures of autochthonous non*-Saccharomyces* strains (*M. pulcherrima MP*20 and *H. vineae HV*11) with *S. cerevisiae* strain (*SC*45) can generate distinct aromatic profiles of icewine compared to *S. cerevisiae* monocultures. Compared to *MP*20, sequential inoculation of *HV*11 and *SC*45 efficiently increased the production of most of the desired volatiles associated with fruity, flowery, and sweety characteristics, enhancing the aromatic diversity of icewine products. Collectively, our data confirmed the positive enological properties of *H. vineae* in the production of icewine and also demonstrated that using indigenous non*-Saccharomyces* strains is a feasible way to improve aromatic diversity of icewine products as expected by consumers.

## Author contributions

C-QD and G-LY designed the experiments. J-YS and B-QZ conducted the experiments. B-QZ, J-YS, and G-LY analyzed the experimental data. G-LY and B-QZ wrote the paper.

### Conflict of interest statement

The authors declare that the research was conducted in the absence of any commercial or financial relationships that could be construed as a potential conflict of interest.

## Footnotes

^1^Available online at: http://www.vqaontario.com/Regulations/Standards (Accessed October 1, 2014).

^2^*GB T25504-2010, Icewine*. Beijing: China Standard Press.
