# Supplementary material for: Use of Indigenous Hanseniaspora vineae and Metschnikowia pulcherrima Co-fermentation With Saccharomyces cerevisiae to Improve the Aroma Diversity of Vidal Blanc Icewine
Source: Front Microbiol. 2018 Oct 22;9:2303. doi: 10.3389/fmicb.2018.02303 (PMC6204404; doi:10.3389/fmicb.2018.02303)
Supplement: Supplementary file 1 [file Table_1.DOCX]

Supplementary Material

**Use of Indigenous *Hanseniaspora vineae* and *Metschnikowia pulcherrima* Co-fermentation with *Saccharomyces cerevisia****e* **to Improve the Aroma Diversity of Vidal blanc Icewine**

**Bo-Qin Zhang^1,2^, Jing-Yun Shen^1,2^, Chang-Qing Duan^1,2^, Guo-Liang Yan^1,2^***

*** *Author for correspondence (Tel: +86-10-62737039; Fax: +86-10-62738658; E-mail:*** [*glyan@cau.edu.cn*](mailto:glyan@cau.edu.cn)***)***

# 1. Supplementary Tables

**TABLE S1 |** **The main enological characteristics of *M. pulcherrima* *MP*20 and *H. vineae* *HV*11**

| Yeast code | Tolerance | |  | SO_2_ resistance ^c^（ppm） | | | |  | Enzymatic activities | |  | H_2_S production ^f^ |
| --- | --- | --- | --- | --- | --- | --- | --- | --- | --- | --- | --- | --- |
|  | High sugar ^a^ | Low temperature ^b^ |  | 25 | 50 | 75 | 100 |  | Protease ^d^ | β-Glucosidase ^e^ |  |  |
| *HV*11 | ＋ | ＋ |  | ＋ | ＋ | ＋ | － |  | ＋ | ＋ |  | 4 |
| *MP*20 | ＋ | ＋ |  | ＋ | ＋ | ＋ | ＋ |  | － | ＋ |  | 3 |

*^a^ The isolated strains (1.0×10^6^ CFU/mL) were inoculated in 50 mL flask with a simple synthetic must described by Ivorra et al. (1999) containing 400 g/L glucose and cultured at 15℃ for 10 d.*

*^b^ The isolated strains (1.0×10^6^ CFU/mL) were inoculated in 50 mL flask with a simple synthetic must Ivorra et al. (1999) and cultured at 10℃ for 10 d.*

*^c^ The SO_2_ resistance was evaluated on YPD medium buffered at pH 3 (with citrate-phosphate buffer), added with increasing doses of K_2_S_2_O_5_.An arbitrary scale was used, with 25, 50, 75, 100 mg/L free SO_2_, respectively. (Domizio et al. 2011)*

*^d^ Extracellular protease production was determined by replica plating yeast colonies onto YPD plates containing 2% casein. The plates were incubated for 5 days at 25℃ and a clear zone around the colony identified protease activity. (Strauss et al. 2001)*

*^e^ The β-glucosidase activity was evaluated as described by Rosi et al. (1994) on agar plates containing arbutin (hydroquinone-β-D-glucopyranoside) as substrate. Strains with this activity hydrolyze the substrate, and dark brown colour develops in the agar.*

*^f^ The H_2_S production of the yeast strains was evaluated on the basis of their colony color (browning degree 0-5) on Bismuth Sulfite Glucose Glycine Yeast (BIGGY) Agar (Sipiczki et al. 2001). 0: no color; 5: strong brown.*

**TABLE S2 | The odor-active compounds detected in our work with their linear fit, R^2^ value, linear rang and aroma characteristics.**

| Compounds | Linear fit | R^2^ value | Linear rang (μg/L) | Odor threshold(μg/L) | Odor characteristics ^n^ | Aroma series |
| --- | --- | --- | --- | --- | --- | --- |
| Isobutanol | y= 61345.90x+ 6448.64 | 0.999 | 82.59-338300.00 | 40000^a^ | Alcohol, solvent, green, bitter | Herbaceous, Chemical |
| Isopentanol | y=8612.26x+2378.55 | 0.989 | 65.12-267080.00 | 30000^a^ | Solvent, alcohol, nail polish | Fatty, Chemical |
| 1-Octen-3-ol | y=232.29x-2.82 | 0.987 | 0.11-1650.01 | 3^e^ | Mushroom | Chemical |
| 2-Phenylethanol | y=2466.51x-38.29 | 0.996 | 4.95-82100.00 | 10000^a^ | Rose, honey | Sweet, Floral |
| 2,3-Butanediol | y=90927.61x+3457.84 | 0.997 | 64.52-264300.00 | 150000^j^ | Fruity, sweet, butter | Fruity, Sweet, Fatty |
| 1-Hexanol | y=781.77x+35.69 | 0.994 | 1.23-20210.00 | 8000^a^ | Herbaceous, grass, woody | Herbaceous |
| (Z)-3-Hexen-1-ol | y=5042.20x+49.18 | 0.989 | 8.15-2087.50 | 1000^k^ | Herbaceous, green, bitter, fatty | Herbaceous, Fatty |
| (E)-3-Hexen-1-ol | y=600.90x+3.11 | 0.989 | 0.22-912.00 | 1000^f^ | Herbaceous, green | Herbaceous |
| 1-Octanol | y=286.21x+0.40 | 0.995 | 0.11-1650.00 | 40^d^ | Jasmine, lemon | Floral, Fruity |
| 2-Octanol | y=110.69x+0.95 | 0.989 | 0.18-304.00 | 120^g^ | - | - |
| Benzyl alcohol | y=5010.36x-7.32 | 0.983 | 0.71-2930.00 | 200000^k^ | Roasted, sweet, fruity | Sweet, Fruity |
| 1-Decanol | y=25.50x-1.22 | 0.998 | 0.05-860.00 | 400^b^ | - | - |
| 2-Nonanol | y=48.14x-0.47 | 0.997 | 0.06~1000.00 | - | - | - |
| 2-Heptanol | y=0.04x+0.02 | 0.984 | 0.17~351.00 | 200^h^ | Fruity, musty, mushroom | Fruity, Chemical |
| Dodecanol | y=113.79x-12.88 | 0.992 | 0.15~645.00 | - | - | - |
| (E)-2-Octen-1-ol | y=340.90x+0.60 | 0.998 | 0.16~668.00 | - | - | - |
| 2-Ethyl-1-hexanol | y=781.77x+35.69 | 0.994 | 1.23-20210.00 | - | - | - |
| Ethyl acetate | y=240.71x+348.48 | 0.984 | 18.91-4854.68 | 7500^a^ | Pineapple, varnish, balsamic | Fruity, Chemical |
| Ethyl butanoate | y=2048.99x+77.39 | 0.986 | 0.34-5540.01 | 20^a^ | Banana, pineapple, strawberry | Fruit |
| Ethyl hexanoate | y=144.71x-276.62 | 0.997 | 4.94-20220.22 | 5^a^ | Banana, green apple | Fruit |
| Ethyl octanoate | y=67.43x-549.61 | 0.987 | 5.11-20920.21 | 2^a^ | Sweet, floral, fruity, banana, pear | Sweet, Floral, Fruity |
| Ethyl decanoate | y=50.41x-482.86 | 0.988 | 5.01-20520.01 | 200^b^ | Fruity, fatty, pleasant | Fruity, Fatty |
| 2-Phenethyl acetate | y=106.20x+3.64 | 0.995 | 0.12-1140.20 | 250^a^ | Fruity, rose | Fruity, Floral |
| Isoamyl acetate | y=15.16x-3.59 | 0.999 | 0.61～1250.00 | 160 ^k^ | Banana | Fruity |
| Ethyl phenylacetate | y=14.91x+2.23 | 0.996 | 0.84-432.00 | 250^c^ | - | - |
| Hexyl acetate | y=137.09x+18.25 | 0.991 | 0.29-1207.51 | 670^f^ | Apple, cherry, pear, floral | Fruity |
| Ethyl heptanoate | y=26.26x+1.01 | 0.994 | 0.03～464.11 | - | - | - |
| Ethyl lactate | y=4.65x+1.73 | 0.989 | 12.12～200000.12 | 154636^b^ | Fruity, butter | Fruity, Sweet, Fatty |
| Ethyl dodecanoate | y=31.63x-68.69 | 0.992 | 0.65-2665.00 | 1500^h^ | Oily, fatty, fruity | Fruity, Fatty |
| Butyl acetate | y=2048.99x+77.39 | 0.986 | 0.34-5540.01 | - | - | - |
| Isobutyl acetate | y=15.16x-3.59 | 0.999 | 0.61～1250.00 | - | - | - |
| Isoamyl octanoate | y=8.11x+0.02 | 0.994 | 0.02-324.00 | 125^b^ | Sweet, fruity, cheese, cream | Sweet, Fruity, Fatty |
| Methyl caprylate | y=17.18x+2.23 | 0.995 | 0.09-1560.00 | 200^g^ | Citrus | Fruity |
| Methyl salicylate | y=15.16x-3.59 | 0.999 | 0.61～1250.00 | - | - | - |
| Diethyl succinate | y=1323.67x+162.93 | 0.996 | 5.11-20940.00 | 1200000^i^ | Fruity, melon | Fruity |
| Linalool | y=46.57x-3.59 | 0.992 | 0.04-179.00 | 25^b^ | Floral | Sweet, Floral |
| Geraniol | y=11167.97x+348.48 | 0.987 | 1.15-4575.75 | 30^a^ | Lemon, geranium | Floral |
| *cis*-Rose oxide | y=18.05x+0.21 | 0.994 | 3.06-196.00 | 0.2^a^ | Lychee | Floral |
| β-Citronellol | y=7.66x+14.20 | 0.987 | 0.46-236.00 | 100^a^ | Rose | Floral |
| 4-Terpineol | y=201.40x-0.64 | 0.995 | 0.12-260.00 | 5000^f^ | - | - |
| α-Terpineol | y=13.58x+0.45 | 0.994 | 0.03-122.00 | 250^b^ | Lilac, floral, sweet | Sweet, Floral |
| β-Damascenone | y=2522.59x+19.59 | 0.984 | 5.07-162.5 | 0.05^m^ | Sweet, exotic, floral, stewed apple | Fruity, Sweet, Floral |
| Phenylacetaldehyde | y=2799.73x-605.96 | 0.984 | 2.61-21350.00 | 1^l^ | Floral, honey | Floral, Sweet |
| Cymbidium | y=6.44x+3.67 | 0.995 | 0.05-210.00 | - | - | - |
| Styrene | y=33.76x-4.60 | 0.998 | 0.25-1022.00 | - | - | - |
| Naphthalene | y=240.71x-271.84 | 0.988 | 0.03-121.00 | - | - | - |
| Geranyl acetate | y=11167.97x+348.48 | 0.987 | 1.15-4575.75 | - | - | - |
| Terpinolene | y=11.30x+6.69 | 0.997 | 0.05~240.00 | - | - | - |
| Phenol | y = 24839.54x＋1.02 | 0.974 | 1.15-4575.75 | - | - | - |
| Pentanoic acid | y=9361.39x-6.39 | 0.995 | 0.87~14360.00 | - | - | - |
| Heptanoic acid | y=2881.32x+157.50 | 0.992 | 1.15~4730.00 | - | - | - |

*^a^ Guth (1997); ^b^ Ferreira et al. (2000); ^c^ López et al. (2002); ^d^ Jørgensen et al. (2001)J; ^e^ La Guerche et al.( 2006); ^f^ Franco et al. (2004); ^g^ Bao and Zhang (2010); ^h^ Tao and Zhang (2010); ^i^ Peinado et al. (2006); ^j^ Sánchezpalomo et al. (2010); ^k^ Peinado et al. (2004); ^l^ Pineau et al. (2007); ^m^ Gomezmiguez et al. (2007); ^n^ Cai et al. (2014)*.

**REFERENCES**

Bao, J., and Zhang, Z. W. (2010). Volatile compounds of young wines from Cabernet Sauvignon, Cabernet Gernischet and Chardonnay varieties grown in the Loess Plateau region of China. *Molecules* 15, 9184-9196.

Cai, J., Zhu, B.Q., Wang, Y.H., Lu, L., Lan, Y.B., Reeves, M.J., et al. (2014). Influence of pre-fermentation cold maceration treatment on aroma compounds of Cabernet Sauvignon wines fermented in different industrial scale fermenters. *Food Chem.* 154, 217-229.

Domizio, P., Romani, C., Lencioni, L., Comitini, F., Gobbic, M., Mannazzu, I., et al. (2011). Outlining a future for non-*Saccharomyces* yeasts: selection of putative spoilage wine strains to be used in association with *Saccharomyces cerevisiae* for grape juice fermentation. *Int. J. Food Microbiol.* 147, 170-180.

Ferreira, V., Lopez, R., and Cacho, J. F. (2000). Quantitative determination of the odorants of young red wines from different grape varieties. *J. Sci. Food Agri.* 80, 1659-1667.

Franco, M., Peinado, R.A., Medina, M., and Moreno, J. (2004). Off-vine grape drying effect on volatile compounds and aromatic series in must from Pedro Ximénez grape variety. *J. Agric. Food Chem.* 52, 3905-3910.

Gomezmiguez, M.J., Cacho, J.F., Ferreira, V., Vicario, I.M., and Heredia, F.J. (2007). Volatile components of Zalema white wines. *Food Chem.* 100, 1464-1473.

Guth, H. (1997). Quantification and sensory studies of character impact odorants of different white wine varieties. *J. Agric. Food Chem.* 45, 3027-3032.

Ivorra, C., Perez-Ortin, J.E., and Del Olmo, M. (1999) An inverse correlation between stress resistance and stuck fermentations in wine yeasts. A molecular study. *Biotechnol. Bioeng*. 64, 698-708.

Jørgensen, L.V., Huss, H.H., and Dalgaard, P. (2001). Significance of volatile compounds produced by spoilage bacteria in vacuum-packed cold-smoked salmon (Salmo salar) analyzed by GC-MS and multivariate regression. *J. Agric. Food Chem.* 49, 2376-2381.

La Guerche, S., Dauphin, B., Pons, M., Blancard, D., and Darriet, P. (2006). Characterization of some mushroom and earthy off-odors microbially induced by the development of rot on grapes. *J. Agric. Food Chem.* 54, 9193-9200.

López, R., Aznar, M., Cacho, J., and Ferreira, V. (2002). Determination of minor and trace volatile compounds in wine by solid-phase extraction and gas chromatography with mass spectrometric detection. *J. Chromatogr. A* 966, 167-177.

Peinado, R.A., Mauricio, J.C., and Moreno, J. (2006). Aromatic series in sherry wines with gluconic acid subjected to different biological aging conditions by *Saccharomyces cerevisiae* var. capensis. *Food Chem.* 94, 232-239.

Peinado, R.A., Moreno, J., Bueno, J.E., Moreno, J.A., and Mauricio, J.C. (2004). Comparative study of aromatic compounds in two young white wines subjected to pre-fermentative cryomaceration. *Food Chem.* 84*,* 585-590.

Pineau, B., Barbe, J.C., Van, L.C., and Dubourdieu, D. (2007). Which impact for beta-damascenone on red wines aroma? *J. Agri. Food Chem.* 55, 4103-4108.

Rosi I., Vinella M., and Domizio, P. (1994) Characterization of beta-glucosidase activity in yeasts of oenological origin. *J. Appl. Bacteriol.* 77, 519-527.

Sánchezpalomo, E., Garcíacarpintero, E.G., Alonsovillegas, R., and Gonzálezviñas, M.A. (2010). Characterization of aroma compounds of Verdejo white wines from the La Mancha region by odour activity values. *Flavour Frag. J.* 25, 456-462.

Sipiczki, M., Romano, P., Lipani, G., Miklos, I., and Antunovic, Z. (2001). Analysis of yeasts derived from natural fermentation in a Tokaj winery. *Anton. Leeuw. Int. J. G*. 79, 97-105.

Strauss, M.L.A., Jolly, N.P., Lambrechts, M.G., and Rensburg, P. (2001). Screening for the production of extracellular hydrolytic enzymes by non-*Saccharomyces* wine yeasts. *J. Appl. Microbiol*. 91, 182-190

Tao, Y., and Zhang, L. (2010). Intensity prediction of typical aroma characters of cabernet sauvignon wine in Changli County (China). *LWT - Food Sc. Technol.* 43, 1550-1556.

**TABLE S3 | Fifty-two volatile aroma compounds (µg/L) in icewines obtained with four *S. cerevisiae* yeasts and mixed inoculation of *SC*45 and non-*Saccharomyces* after alcoholic fermentation.**

| Compounds | XR | R2 | *SC*42 | *SC*45 | SI-*MP*20/*SC*45 | SE-2-*MP*20/*SC*45 | SE-4-*MP*20/*SC*45 | SI-*HV*11/*SC*45 | SE-2-*HV*11/*SC*45 | SE-4-*HV*11/*SC*45 |
| --- | --- | --- | --- | --- | --- | --- | --- | --- | --- | --- |
| **C6 alcohol** |  |  |  |  |  |  |  |  |  |  |
| 1-Hexanol | 1163.1±29.0d | 1103.5±1.2bc | 1089.2±29.7b | 1095.6±1.3bc | 1043.4±8.2a | 1026.4±19.9a | 1036.7±4.3a | 1128.2±7.0c | 1080.4±5.4b | 1172.6±10.4d |
| (Z)-3-Hexen-1-ol | 166.3±0.6a | 153.4±10.1a | 167.8±7.5ab | 154.2±6.9a | 163.9±17.1a | 171.9±1.4ab | 174.8±9.3ab | 177.8±2.4ab | 186.8±10.8ab | 201.4±35.2b |
| (E)-3-Hexen-1-ol | 183.5±5.6a | 175.7±23.3a | 190.5±23.3a | 170.6±2.5a | 174.1±15.5a | 173.0±7.7a | 175.7±0.5a | 184.0±7.4a | 197.9±38.3a | 237.7±8.7b |
| ***Total of C6 alcohols*** | **1512.9±24.1e** | **1432.6±32.2bc** | **1447.5±60.54bc** | **1420.4±10.7b** | **1381.5±40.8a** | **1371.3±13.6a** | **1387.3±14.2a** | **1490.0±16.8d** | **1465.1±43.7cd** | **1611.8±54.4f** |
| **Higher alcohols** |  |  |  |  |  |  |  |  |  |  |
| Isobutanol | 68448.6±7.1g | 67829.7±11.6f | 59640.7±38.6b | 55943.8±9.0a | 87571.6±10.8j | 62209.2±0.6c | 66621.1±30.4e | 83820.9±17.4i | 64665.8±39.1d | 71131.8±18.3h |
| Isopentanol | 48084.5±42.5i | 42758.8±5.1e | 40830.5±49.1a | 41663.3±8.4c | 43591.3±4.9f | 45394.8±24.9h | 44436.6±3.4g | 50179.0±7.9j | 41003.3±2.0b | 41961.5±0.6d |
| 1-Octen-3-ol | 164.5±4.4a | 159.0±11.5a | 160.9±0.3a | 158.4±4.6a | 160.8±11.7a | 167.8±4.1a | 172.2±15.4a | 172.0±1.6a | 168.2±18.1a | 179.7±22.8a |
| 2-Phenylethanol | 4008.3±11.5d | 2313.3±0.7a | 4520.4±38.3f | 3195.8±12.4b | 4113.9±5.8e | 5001.3±1.3g | 3519.0±1.7c | 7860.6±14.0h | 9816.9±2.4i | 12229.8±13.9j |
| 2,3-Butanediol | 111000.2±10.3e | 104000.3±9.55a | 104950.0±4.3b | 129531.1±5.2j | 105872.2±5.8d | 113000.7±17.3g | 105342.2±13.1c | 112430.5±9.9f | 113789.7±4.1h | 113998.6±9.9i |
| 1-Octanol | 10.5±1.3a | 30.7±1.1e | 27.2±0.1e | 31.0±6.7e | 30.4±3.4e | 26.5±1.8de | 26.5±2.0de | 19.3±2.5bc | 15.3±0.4ab | 12.5±4.8ab |
| 2-Octanol | 16.4±1.4ab | 14.8±1.3ab | 15.8±0.1ab | 14.8±0.7ab | 13.8±1.3ab | 12.9±0.4a | 12.9±1.8a | 16±0.6ab | 15.4±2.5ab | 17.9±3.5b |
| Benzyl alcohol | 72.2±27.7ab | 29.5±5.0a | 81.0±22.2b | 65.8±0.9ab | 53.2±9.5ab | 90.6±22.9b | 64.4±5.2ab | 152.7±1.1b | 137.9±20.2b | 218.0±42.9d |
| 1-Decanol | 2.3±0.1d | 1.8±0.0bc | 1.9±0.0a | 1.7±0.3ab | 1.4±0.0a | 1.4±0.0a | 1.4±0.0a | 1.4±0.0a | 1.4±0.0a | 1.4±0.0a |
| 2-Nonanol | 18.2±0.0a | 18.4±0.1a | 18.9±0.9a | 18.5±0.3a | 18.1±0.0a | 18.8±1.0a | 18.2±0.1a | 18.0±0.1a | 18.8±1.0a | 18.6±1.3a |
| 2-Heptanol | 7.0±3.3ab | 10.9±1.0ab | 6.1±0.1a | 7.6±1.9ab | 6.3±0.5a | 10.7±3.5ab | 10.2±3.6ab | 8.9±0.7ab | 10.8±1.5ab | 11.5±0.6b |
| Dodecanol | 8.1±0.1a | 8.1±0.0a | 8.1±0.0a | 8.1±0.1a | 8.1±0.0a | 8.1±0.1a | 8.1±0.0a | 8.1±0.0a | 8.2±0.1a | 8.3±0.1a |
| (E)-2-Octen-1-ol | 1.9±0.8a | 2.3±0.6a | 5.1±6.6a | 1.4±0.5a | 1.3±0.4a | 0.7±0.5a | 0.5±0.5a | 1.1±1.2a | 2.0±0.1a | 5.7±1.6a |
| 2-Ethyl-1-hexanol | 19.4±0.1a | 20.2±0.9a | 18.5±0.0a | 19.3±1.6a | 17.3±0.2a | 17.9±1.0a | 24.3±10.1a | 18.0±0.5a | 18.0±1.3a | 17.1±1.8a |
| ***Total of higher alcohols*** | **231861.8±87.1c** | **217197.5±31.7a** | **210285.0±78.0a** | **230660.4±17.4c** | **241459.5±9.3d** | **225960.7±0.7b** | **220257.5±18.1b** | **254706.1±9.1e** | **229671.1±33.6bc** | **239811.8±58.5d** |
| **Acetate esters** |  |  |  |  |  |  |  |  |  |  |
| Ethyl acetate | 7045.6±30.8c | 7871.2±0.1h | 7441.5±9.7e | 5626.4±8.0a | 8762.7±13.3j | 7147.5±43.7d | 7556.6±40.5f | 8532.3±7.4i | 6946.4±1.0b | 7785.7±8.6g |
| 2-Phenethyl acetate | 3103.6±42.7e | 2017.5±10.7a | 2774.7±21.1c | 2590.8±11.4b | 2878.8±25.8d | 3446.0±35.1g | 3197.4±47.7f | 4556.4±41.9h | 8341.6±0.1j | 6716.0±15.4i |
| Isoamyl acetate | 1209.0±0.9f | 863.3±2.5a | 993.0±1.9bc | 963.6±27.9b | 1031.0±10.4cd | 1075.9±3.2d | 1062.6±2.2d | 1129.1±31.3e | 1048.0±41.4d | 1047.0±2.0d |
| Hexyl acetate | 169.5±0.7g | 121.5±0.5c | 140.6±14.8e | 129.2±0.2d | 81.9±1.8b | 158.5±3.1e | 148.0±3.8ef | 142.2±2.4e | 72.5±5.4a | 126.7±3.7cd |
| Butyl acetate | 10.1±5.6b | 2.0±1.6a | 6.5±1.7ab | 8.5±4.7ab | 16.0±0.0c | 9.7±3.1b | 12.3±1.3b | 6.9±2.8ab | 5.4±1.2ab | 7.1±1.8ab |
| Isobutyl acetate | 70.7±3.8ab | 68.7±2.1a | 69.7±1.9ab | 66.7±2.1a | 82.1±2.2c | 71.8±0.8ab | 71.2±0.2ab | 74.6±2.6b | 71.7±3.9ab | 72.4±0.4ab |
| ***Total of acetate esters*** | **11608.5±4.1c** | **10944.2±17.5b** | **11426.0±27.8c** | **9385.3±5.7a** | **12996.4±1.8d** | **11909.3±1.5c** | **12048.1±85.1d** | **14441.5±0.9e** | **16485.6±50.8g** | **15755.1±10.8f** |
| **Ethyl esters** |  |  |  |  |  |  |  |  |  |  |
| Ethyl butanoate | 264.4±40.0bc | 224.3±37.7ab | 302.1±1.5c | 233.4±26.7ab | 316.1±5.8c | 246.2±5.2ab | 213.9±23.9ab | 249.0±10.9ab | 199.6±11.3a | 233.9±20.3ab |
| Ethyl hexanoate | 2543.8±32.9d | 2261.9±13.9b | 2361.6±26.4a | 3119.9±35.1f | 2814.4±6.4e | 2136.8±8.5a | 2249.8±6.1b | 2240.4±24.5b | 2844.8±10.9e | 2569.9±40.1d |
| Ethyl octanoate | 574.1±5.6d | 482.9±1.0c | 681.7±19.7f | 1909.9±2.8i | 292.5±4.8a | 867.4±4.5h | 566.9±9.2d | 720.5±12.4g | 621.4±11.2e | 420.1±7.2b |
| Ethyl decanoate | 523.5±21.7b | 542.1±7.5b | 604.7±49.8c | 719.0±16.4e | 479.9±20.1a | 795.7±2.5f | 656.9±9.0d | 608.9±1.8c | 548.5±2.9b | 680.9±5.2de |
| Ethyl phenylacetate | 40.7±0.0ab | 40.7±0.0ab | 40.7±0.0ab | 40.4±0.3a | 43.8±4.1b | 40.9±0.1ab | 40.8±0.1ab | 41.0±0.2ab | 41.4±0.3ab | 41.3±0.0ab |
| Ethyl heptanoate | 6.7±0.8e | 2.6±1.4abc | 2.3±0.3abc | 12.6±0.6f | 1.2±0.4a | 3.9±0.7cd | 5.9±0.8e | 2.9±0.0bc | 5.1±0.1de | 2.1±0.6ab |
| Ethyl lactate | 1907.1±36.3e | 1361.3±19.7b | 1577.5±24.8c | 1068.9±18.1a | 1538.2±44.7c | 1349.4±6.4b | 1526.5±2.0c | 1762±18.1d | 1770.3±3.1d | 1717.2±17.0d |
| Ethyl dodecanoate | 97.3±4.5ab | 100.6±1.5ab | 106.9±2.9abc | 108.8±37.4abc | 87.9±5.8a | 125.1±4.3bc | 123.8±1.4bc | 122.9±2.2bc | 133.4±2.6c | 121.4±8.7bc |
| ***Total of ethyl esters*** | **5957.7±74.6cd** | **5016.3±49.1a** | **5677.5±20.1bc** | **7212.9±60.4e** | **5574.0±33.3b** | **5565.4±3.0b** | **5384.3±17.5ab** | **5747.6±15.5c** | **6164.5±30.2d** | **5786.8±31.9c** |
| **Other esters** |  |  |  |  |  |  |  |  |  |  |
| Isoamyl octanoate | 9.3±0.2a | 9.3±0.0a | 9.1±0.1a | 9.2±1.6a | 9.5±0.2a | 8.4±0.1a | 8.4±0.0a | 8.8±0.1a | 8.6±0.1a | 8.5±0.2a |
| Methyl caprylate | 8.4±0.4a | 8.3±0.3a | 8.0±0.4a | 8.5±2.3a | 9.2±0.4a | 7.4±0.1a | 7.4±0.1a | 7.6±0.2a | 7.9±0.5a | 7.8±0.4a |
| Methyl salicylate | 6.9±0.0a | 6.9±0.1a | 6.9±0.0a | 6.9±0.1a | 6.9±0.0a | 6.9±0.0a | 6.9±0.0a | 6.8±0.1a | 6.8±0.0a | 6.8±0.1a |
| Diethyl succinate | 1498.1±7.1bcd | 1535.6±6.7f | 1526.7±10.3def | 1547.7±24.6f | 1534.3±5.5ef | 1505.0±12.4cde | 1526.6±3.7def | 1490.1±23.1abc | 1473.1±2.7ab | 1465.2±2.7a |
| ***Total of all esters*** | **19088.9±62.9d** | **17520.5±59.7a** | **18654.2±37.2c** | **18170.6±37.6b** | **20130.3±29.1e** | **19002.4±10.8d** | **18981.6±71.4d** | **21702.5±6.4f** | **24146.5±83.4h** | **23030.2±46.0g** |
| **Terpenes** |  |  |  |  |  |  |  |  |  |  |
| Linalool | 42.4±0.2ab | 41.5±0.5ab | 40.9±0.4a | 42.0±0.9ab | 41.6±1.3ab | 43.5±0.0bc | 44.6±0.7cd | 45.4±0.7cde | 46.6±0.2de | 47.2±2.3e |
| Geraniol | 1924.0±1.2ab | 1921.7±0.1a | 1924.4±0.2ab | 1936.4±17.5b | 1921.1±1.0a | 1925.7±0.8ab | 1924.8±1.5ab | 1926.3±2.9ab | 1930.5±1.5ab | 1931.3±0.1ab |
| *cis*-Rose oxide | 14.9±1.8d | 9.9±0.9b | 7.9±0.4a | 14.0±0.4c | 16.0±0.9g | 15.3±0.2f | 15.1±0.5e | 16.7±0.4h | 17.2±0.5i | 18.2±0.3j |
| β-Citronellol | 20.3±0.2ab | 19.2±0.3ab | 21.2±0.0abc | 20.2±2.7ab | 17.8±0.2a | 23.1±0.6bc | 22.0±0.6abc | 22.0±1.7abc | 25.6±4.4c | 25.4±1.0c |
| 4-Terpineol | 11.0±1.1a | 11.1±2.7a | 14.0±2.9a | 37.0±34.4a | 9.7±5.0a | 16.9±4.7a | 14.4±0.1a | 18.3±0.6a | 23.3±2.8a | 22.7±4.8a |
| α-Terpineol | 0.8±0.1cd | 0.8±0.0d | 0.8±0.1d | 0.8±0.0d | 0.7±0.1cd | 0.7±0.1bcd | 0.6±0.0bc | 0.5±0.1b | 0.2±0.2a | 0.1±0.1a |
| ***Total of terpenes*** | **2013.3±4.7abc** | **2004.3±1.3a** | **2009.2±2.0ab** | **2050.3±43.2c** | **2007.0±5.4ab** | **2025.1±5.8abc** | **2021.5±9.4abc** | **2029.2±7.0abc** | **2043.2±3.5abc** | **2044.9±4.2bc** |
| **Others** |  |  |  |  |  |  |  |  |  |  |
| β-Damascenone | 42.3±1.6b | 41.9±0.9b | 40.0±1.47a | 48.1±1.19c | 40.9±0.5ab | 55.6±0.6d | 62.7±0.9e | 67.1±1.8f | 101.7±3.09h | 96.5±1.0g |
| Phenylacetaldehyde | 1.6±1.5ab | 2.5±0.7ab | 1.4±1.6ab | 4.5±2.4ab | 0.3±0.1a | 2.8±1.8ab | 4.6±0.8ab | 3.2±0.7ab | 10.3±9.6b | 5.1±5.8ab |
| Cymbidium | 5.8±1.5d | 6.4±2.5de | 7.2±0.7de | 9.4±1.5e | 1.2±0.3ab | 5.1±0.2cd | 4.2±2.2bcd | 0.5±0.1a | 0.6±0.7a | 2.1±1.1abc |
| Styrene | 129.4±0.0d | 128.5±0.2bcd | 127.3±0.5ab | 129.7±0.6d | 126.8±0.8a | 129.1±0.1bcd | 129.1±0.1cd | 129.0±0.1bcd | 128.6±0.1bcd | 127.6±1.9abc |
| Naphthalene | 11.5±0.0cd | 11.5±0.0bcd | 11.4±0.1bc | 11.6±0.1d | 11.3±0.0a | 11.5±0.1bcd | 11.5±0.0bc | 11.4±0.0bc | 11.4±0.0ab | 11.3±0.1a |
| Geranyl acetate | 47.8±11.1ab | 56.4±0.1b | 46.4±1.1ab | 25.5±19.0a | 71.4±14.8bc | 57.3±0.3b | 61.6±17.3bc | 84.0±0.3cd | 95.8±1.4d | 106.5±8.5d |
| Terpinolene | 17.9±0.2de | 17.8±0.0de | 18.2±0.1ef | 18.5±0.3f | 17.1±0.3bc | 17.8±0.0de | 17.5±0.2cd | 17.1±0.2bc | 16.7±0.1ab | 16.4±0.3a |
| Phenol | 4.8±1.7a | 20.2±7.8a | 5.0±0.51a | 3.2±2.4a | 3.8±0.9a | 6.0±2.5a | 23.1±25.0a | 19.4±16.5a | 11.2±6.6a | 8.0±1.4a |
| Pentanoic acid | 327.9±35.5a | 338.3±0.7ab | 357.4±7.6ab | 339.3±25.5ab | 319.3±23.4a | 329.8±38.2ab | 346.9±0.6ab | 356.7±12.8ab | 360.3±5.3ab | 379.3±3.5b |
| Heptanoic acid | 93.8±1.1a | 96.0±0.4a | 101.0±2.2ab | 96.3±7.1a | 94.9±1.1a | 99.0±3.2ab | 97.7±1.1ab | 100.9±6.3ab | 105.1±3.6bc | 112.0±1.1c |

*XR : S. cerevisiae XR pure fermentation; R2: S. cerevisiae R2 pure fermentation; SC42: S. cerevisiae SC42 pure fermentation; SC45: S. cerevisiae SC45 pure fermentation; SI-MP20/SC45: Simultaneous inoculation of MP20 and SC45; SE-2-MP20/SC45: Sequential inoculation of MP20 followed by SC45 after two days; SE-4-MP20/SC45: Sequential inoculation of MP20 followed by SC45 after four days; SI- HV11/SC45: Simultaneous inoculation of HV11 and SC45; SE-2- HV11/SC45:Sequential inoculation of HV11 followed by SC45 after two days; SE-4- HV11/SC45: Sequential inoculation of HV11 followed by SC45 after four days.*

*Sixteen odor active compounds (OVA>1) were underlined. Values are given as mean ± standard deviation of two biological replicates and three HPLC detection runs. Data with different letters (a, b, c, d, e, f, g, h, i) within each column are different according to Duncan tests (0.05%).*
